# Selective connectivity enhances storage capacity in attractor models of memory function

**DOI:** 10.3389/fnsys.2022.983147

**Published:** 2022-09-15

**Authors:** Facundo Emina, Emilio Kropff

**Affiliations:** ^1^Universidad de Buenos Aires, Facultad de Ciencias Exactas y Naturales, Departamento de Física, Buenos Aires, Argentina; ^2^Leloir Institute—IIBBA/CONICET, Buenos Aires, Argentina

**Keywords:** autoassociative memory, Hopfield network, structural plasticity, connectivity optimization, storage capacity, computational models, attractor dynamics

## Abstract

Autoassociative neural networks provide a simple model of how memories can be stored through Hebbian synaptic plasticity as retrievable patterns of neural activity. Although progress has been made along the last decades in understanding the biological implementation of autoassociative networks, their modest theoretical storage capacity has remained a major constraint. While most previous approaches utilize randomly connected networks, here we explore the possibility of optimizing network performance by selective connectivity between neurons, that could be implemented in the brain through creation and pruning of synaptic connections. We show through numerical simulations that a reconfiguration of the connectivity matrix can improve the storage capacity of autoassociative networks up to one order of magnitude compared to randomly connected networks, either by reducing the noise or by making it reinforce the signal. Our results indicate that the signal-reinforcement scenario is not only the best performing but also the most adequate for brain-like highly diluted connectivity. In this scenario, the optimized network tends to select synapses characterized by a high consensus across stored patterns. We also introduced an online algorithm in which the network modifies its connectivity while learning new patterns. We observed that, similarly to what happens in the human brain, creation of connections dominated in an initial stage, followed by a stage characterized by pruning, leading to an equilibrium state that was independent of the initial connectivity of the network. Our results suggest that selective connectivity could be a key component to make attractor networks in the brain viable in terms of storage capacity.

## 1. Introduction

The dynamics and functionality of neural networks, both artificial and biological, are strongly influenced by the configuration of synaptic weights and the architecture of connections. The ability of a network to modify synaptic weights plays a central role in learning and memory. Networks in cortical areas and in the hippocampus are believed to store patterns of activity through synaptic plasticity, making possible their retrieval at a later stage, or equivalently the replay of past network states (Citri and Malenka, 2008; Carrillo-Reid et al., 2016). Autoassociative neural networks have emerged as crucial models to describe this mode of computation by means of attractor dynamics. Memories can be thought of as attractor states that arise as a consequence of configuring synaptic weights following a Hebbian learning rule, where the synapse between two neurons is strengthened or weakened depending on how correlated is their activity (Hopfield, 1982).

A second, less explored way in which interactions between neurons in autoassociative memories can be modified is by adding or deleting connections. Evidence suggests that topological characteristics of biological neural networks are far from random, and might result from a trade-off between energy consumption minimization and performance maximization (Bullmore and Sporns, 2012). Over the course of evolution, what is now the human brain grew by several orders of magnitude in terms of the number of neurons (*N*), but the number of connections per neuron (*c*) has remained rather stable (Assaf et al., 2020). A limitation for increasing *c* is the scaling of the volume and mass associated to it, and the fact that white matter (long-range connections) represents already approximately half of the total mass of the human brain might indicate a tight compromise (Herculano-Houzel et al., 2010). The number of connections, however, is not constant throughout human life. The formation of neuronal connections in the central nervous system is a highly dynamic process, consisting of simultaneous events of elimination and formation of connections (Hua and Smith, 2004). Studies suggest that a common rule in many parts of the brain consists of an initial stage where the creation of connections dominates, peaking during childhood at around 2 years old, and a later reversion stage where pruning dominates until an equilibrium is reached during late adolescence or early adulthood (Huttenlocher, 1979; Lichtman and Colman, 2000; Navlakha et al., 2015). In addition, evidence such as profound changes in dendritic branching in some cortical areas after exposing animals to complex environments and cortical axon remodeling after lesions of the sensory periphery support the idea that structural changes in the wiring diagram might be a vital complement of the learning scheme based on synaptic plasticity (Chklovskii et al., 2004).

Several theoretical variations of the Hopfield network have been proposed to describe the functionality of the hippocampus and other brain areas by means of attractor dynamics, with varying degree of biological plausibility (Amit, 1989; Treves and Rolls, 1991; Kropff and Treves, 2007; Roudi and Latham, 2007). All these variants, however, share a key limitation of the original Hopfield model regarding the number of memories that a network can store and successfully retrieve. Fully connected Hopfield models, for example, can only store a number of patterns equal to a fraction α_*c*_ ~ 0.138 of the connections per neuron. If more memories are stored, a phase transition occurs and the network loses its ability to retrieve any of the stored patterns. Real brains would need to stay far from the transition point to avoid this risk, a constraint under which networks in the human brain, which has *N* ~ 10^11^ neurons but on average only *c* ~ 10^4^ connections per neuron, would be able to store <*p* ~ 10^3^ memories, a rather modest number. Error-correcting iterative algorithms as an alternative technique to set the synaptic weight configuration have been shown to increase the storage capacity up to α_*c*_ = 2 (Forrest, 1988; Gardner, 1988). However, the use of fully connected networks and non-Hebbian modification of synaptic weights limits the applicability of these ideas to model real brains. Random sparse connectivity offers only a relative gain in network performance, allowing the storage capacity to go up to α_*c*_ = 2/π in ultra-diluted networks, where *c* ≪ ln(*N*) (Derrida et al., 1987), or a slightly lower limit in less diluted networks where *c* ≪ *N* (Arenzon and Lemke, 1994). Some studies suggest that selective pruning might be much more effective than random dilution in terms of storage capacity, making it diverge for ultra-diluted networks (Montemurro and Tamarit, 2001) or increasing the memory robustness (Janowsky, 1989).

Autoassociative networks gain an order of magnitude in storage capacity when including a more realistic sparse activity (involving ~ 5−10% of neurons per pattern) (Tsodyks and Feigel'man, 1988; Tsodyks, 1989), although this gain is compensated by similar losses related to the incorporation of other biologically plausible elements in either the modeled neurons or the statistics of the stored data (Kropff and Treves, 2007; Roudi and Latham, 2007). Other models that consider the cortex as a network of networks suggest that a hierarchical strategy does not yield considerable benefits, since the number of bits of information that can be stored per synaptic variable is very similar to that of simpler models (Kropff and Treves, 2005).

What other strategies could have been developed by our brains to increase the storage capacity beyond the limit of hundreds of memories predicted by the Hopfield model? In this work we explore the possibility of introducing modifications to the architecture of connections with the aim of improving the signal-to-noise ratio. This process could be analogous to the formation and pruning of connections that reshapes our brains throughout maturation. In the first section we show through numerical simulations that autoassociative networks are able to increase their storage capacity up to around seven-fold by minimizing the noise. In the second section, we show that if the cost function aims to reinforce the signal rather than minimizing the noise, a gain of up to almost one order of magnitude can be obtained. In the last section we implement an algorithm where connections are constantly added and pruned as the network learns new patterns, showing that it converges to the same connectivity with optimal storage capacity regardless of the starting conditions, and that if initial conditions are of low connectivity it reaches an early maximum followed by a long period of decay, as is the case generally in the human cortex.

## 2. Materials and methods

### 2.1. Hopfield model

#### 2.1.1. Autoassociative network

For simplicity, we utilized a network similar to the one originally proposed by Hopfield, capable of storing information in the form of activity patterns that can be later retrieved by a partial cue. The network consists of *N* recurrently connected neurons, each receiving *c* pre-synaptic connections with no self-connections. The state of neuron *i* at time *t* is represented by $s_{i}^{t}$ and can take two possible values: $s_{i}^{t}=1$ (“active”) or $s_{i}^{t}=-1$ (“inactive”). The activity of the network evolves synchronously at discrete time steps. At each time step neurons receive a local activity field given by the weighted sum of the activity of other neurons


(1) $$\begin{array}{r} h_{i}^{t}=\frac{1}{c}\overset{N}{\underset{j=1}{\sum }}W_{ij}C_{ij}s_{j}^{t}, \end{array}$$

where *W*_*ij*_ represents the synaptic weight between the pre-synaptic neuron *j* and the post-synaptic neuron *i*, *C*_*ij*_ is a binary matrix taking a value of 1 if this physical connection exists and 0 otherwise and *c* is the number of pre-synaptic connections targeting a given neuron, so that $\overset{N}{\underset{\text{j}=1}{\sum }}C_{ij}=c$ for all *i* (*C*_*ii*_ = 0 because there are no self-connections). Since we are interested in studying the effects of adding and removing connections, it is convenient to consider separately a synaptic matrix $\bar{\bar{W}}$ with values that only depend on the stored patterns and a matrix $\bar{\bar{C}}$ that only indicates whether or not the connection exists.

After calculating $h_{i}^{t}$, neurons update their state following a deterministic update rule,


(2) $$\begin{array}{r} s_{i}^{t+1}=\mathrm{sgn}\left(h_{i}^{t}\right), \end{array}$$

where the function sgn(*x*) returns the sign of *x*.

Synaptic weights are computed following a linear Hebbian rule,


(3) $$\begin{array}{r} W_{ij}=\overset{p}{\underset{\mu =1}{\sum }}\xi_{i}^{\mu }\xi_{j}^{\mu }, \end{array}$$

where $\xi_{i}^{\mu }$ represents the state of neuron *i* in pattern μ, from a total amount of *p* stored patterns. In the classic Hopfield model (and in this work), activity patterns come from the random distribution,


(4) $$\begin{array}{r} P\left(\xi_{i}^{\mu }\right)=\frac{1}{2}\delta \left(\xi_{i}^{\mu }-1\right)+\frac{1}{2}\delta \left(\xi_{i}^{\mu }+1\right). \end{array}$$

#### 2.1.2. Storage capacity

The relevant parameter that describes the storage capacity of a network is α = *p*/*c*. A critical value α_*c*_ exists where, if more patterns are loaded, the network suffers a phase transition to an amnesic state. This phase transition can be understood in terms of the local activity field each neuron receives. If the network has *p* patterns ideally stored (i.e., every pattern is exactly a fix point of the network dynamics) and at time *t* the network is initialized so that the state of each neuron corresponds to a given pattern ${\bar{\xi }}^{\nu }$ (i.e., $s_{i}^{t}=\xi_{i}^{\nu }$ for all neurons), then no changes in the neuronal states should occur as a consequence of the update rule in Equation (2), i.e., $s_{i}^{t+1}=s_{i}^{t}=\xi_{i}^{\nu }$. To understand this, the local activity field that neuron *i* receives $h_{i}^{t}\equiv h_{i}^{\nu }$ can be split into a signal term $\xi_{i}^{\nu }$ (resulting from the contributions of stored information corresponding to pattern ν) and a noise term $R_{i}^{\nu }$ (which can be thought of as the contribution of other stored patterns)


(5) $$\begin{array}{l} s_{i}^{t+1}=\mathrm{sgn}(h_{i}^{\nu }),\\ \;h_{i}^{\nu }=\xi_{i}^{\nu }+\frac{1}{c}\sum_{j=1}^{N}\sum_{\mu \neq \nu }^{p}\xi_{i}^{\mu }\xi_{j}^{\mu }\xi_{j}^{\nu }C_{ij}=\xi_{i}^{\nu }+R_{i}^{\nu }. \end{array}$$

If the local field has the same sign as $\xi_{i}^{\nu }$, or equivalently, if the aligned local field is positive for each neuron ($h_{i}^{\nu }\xi_{i}^{\nu }\equiv 1+R_{i}^{\nu }\xi_{i}^{\nu }>0$), then the pattern ${\bar{\xi }}^{\nu }$ will be exactly retrieved. In other words, pattern retrieval depends on whether or not the noise term flips the sign of the aligned local activity field from positive to negative. If connectivity is diluted and random, the aligned local field can be approximated by a random variable following a normal distribution of unitary mean and standard deviation $\sigma \sim \sqrt{p/c}$. Since this standard deviation increases monotonically with the number of stored patterns, there is a critical point beyond which the noise fluctuations are large enough to destabilize all patterns and prevent them from being recovered (i.e., $h_{i}^{\nu }\xi_{i}^{\nu }<0$ for a critical number of neurons).

#### 2.1.3. Basin of attraction

A fundamental characteristic of the attractor states of a Hopfield network is their basin of attraction. It is a quantification of the network's tolerance to errors in the initial state. The basin of attraction depends on the connectivity of the network and the number of patterns stored. In randomly connected networks with low memory load (*p* ≪ *p*_*c*_), every pattern can be retrieved if the cue provided to the network represents at least 50% of the pattern (<50% is a cue for the retrieval of a stable spurious state represented by flipping all elements in the pattern). As the memory load approaches the critical value *p*_*c*_, tolerance to error smoothly weakens.

We studied memory robustness by simulating networks with different connectivity and memory load. For each initial error, we counted the number of patterns the network could successfully retrieve (e.g., initializing the network in pattern ${\bar{\xi }}^{\nu }$ with an error of 0.2 implied that 20% of the neurons initially deviated from the pattern). We studied the percentage of successfully retrieved patterns as a function of error and memory load.

### 2.2. Simulations

Simulations were run in custom made scripts written in MATLAB (RRID:SCR_001622). In all sections, the connectivity matrix $\bar{\bar{C}}$ of each simulated network was initially constructed pseudo-randomly. We selected for each of the *N* neurons its *c* pre-synaptic connections using MATLAB's function *randperm*(*N*−1, *c*), randomly obtaining the *c* indexes among their *N*−1 possible inputs.

In a typical simulation to study storage capacity, we initialized the network in a given pattern ν, i.e., $s_{i}^{0}=\xi_{i}^{\nu }$, and updated the network following Equation (2) for 100 iterations or until the overlap between the pattern and the network state (i.e., $\frac{1}{N}\overset{N}{\underset{j=1}{\sum }}s_{j}^{t}\xi_{j}^{\nu }$) remained constant. After this, the pattern was classified as successfully retrieved if the overlap between the final state of the network and the pattern ν was >0.7 (implying that the attractor associated to the pattern was stable but possibly slightly distorted by interference with other patterns). Note that, as is usual when working close to the storage capacity limit, we did not require patterns to be exact fixed points of the network dynamics in order to be considered as successfully stored. Unless otherwise specified, the storage capacity of the network was defined as the maximum number of patterns for which the network could successfully retrieve all of them, normalized by *c*.

### 2.3. Connectivity optimization

#### 2.3.1. Noise reduction

As described above, the retrieval of memories can be compromised by random fluctuations in the noise term making the aligned local field negative. We asked whether a non-random connectivity matrix could substantially reduce the local noise contribution each neuron received, resulting in an increase in the storage capacity. In order to find an optimal connectivity matrix, we proposed each neuron to select its *c* pre-synaptic connections by minimizing an energy function


(6) $$\begin{array}{r} E_{i}^{0}=\overset{p}{\underset{\nu =1}{\sum }}{\left(\overset{N}{\underset{j=1}{\sum }}\overset{p}{\underset{\mu \neq \nu }{\sum }}\xi_{i}^{\mu }\xi_{j}^{\mu }\xi_{i}^{\nu }\xi_{j}^{\nu }C_{ij}\right)}^{2}\propto \overset{p}{\underset{\nu =1}{\sum }}{\left(R_{i}^{\nu }\right)}^{2}. \end{array}$$

Note that in an ideal connectivity configuration that cancels $E_{i}^{0}$, neuron *i* would receive zero noise during the retrieval of any of the stored patterns. In order to use the number of pre-synaptic connections as a control parameter, we used a fixed *c*, implying that this minimization problem is subject to the constraint $\overset{N}{\underset{j=1}{\sum }}C_{ij}=c$. Other constraints inherent to the nature of the connectivity matrix is that it is binary (*C*_*ij*_∈{0;1}) with *C*_*ii*_ = 0. Thus, the minimization of Equation (6) belongs to the family of quadratic constrained binary optimization problems. To obtain a computationally efficient approximate solution to this problem we implemented an adaptation of the simulated annealing algorithm. We applied independently to each neuron's pre-synaptic connectivity an annealing schedule where temperature *T* was decreased by a factor of 0.99 at each step. For each neuron *i*, we randomly selected two elements in the *i*-*th* row of the connectivity matrix $\bar{\bar{C}}$ and permuted them, which ensured an invariant *c*. The change was always accepted if the cost function $E_{i}^{0}$ decreased ($\text{Δ}E_{i}^{0}<0$), or otherwise with a probability equal to $e^{-\text{Δ}E_{i}^{0}/T}$. Initial temperature was estimated following the method detailed by Yang (2014) and final temperature was set to 10^−4^.

#### 2.3.2. Signal reinforcement

We proposed a second cost function which can be thought of as a generalization of Equation (6). In this case, the aim is not to minimize the noise but instead to reinforce the signal, contributing positively to the aligned local field by making $R_{i}^{\nu }\xi_{i}^{\nu }>0$,


(7) $$\begin{array}{r} E_{i}^{\epsilon }=\overset{p}{\underset{\nu =1}{\sum }}{\left(\overset{N}{\underset{j=1}{\sum }}\overset{p}{\underset{\mu \neq \nu }{\sum }}\xi_{i}^{\mu }\xi_{j}^{\mu }\xi_{i}^{\nu }\xi_{j}^{\nu }C_{ij}-\epsilon \right)}^{2}\propto \overset{p}{\underset{\nu =1}{\sum }}{\left(R_{i}^{\nu }\xi_{i}^{\nu }-\epsilon \right)}^{2}, \end{array}$$

where ϵ is a non-negative parameter. Note that setting ϵ = 0 makes this problem equivalent to the noise reduction scenario.

Results shown in Section 3.2 correspond to optimizations with ϵ = *p* (we refer to the energy function in Equation 7 as $E_{i}^{p}$). This arbitrary choice is justified by the observation in a preliminary analysis that the optimization with this value of ϵ nearly maximized the number of patterns where noise contributed positively to the local field for a wide range of values of *c*, *p*, and *N*.

#### 2.3.3. Online algorithm

As mentioned in Section 1, addition and pruning of connections are features of brain maturation. To gain an insight into the role they could play in a learning network, we also proposed an online optimization algorithm where the incorporation of memories and the modification of the connectivity through signal reinforcement occurred in parallel. Our aims were to understand if a similar improvement in storage capacity could be achieved through this on-line approach and to study the dynamics of connectivity.

Algorithm 1 consisted of a heuristic optimization where each neuron would independently attempt to minimize $E_{i}^{\epsilon }$ (Equation 7) by eliminating and generating input connections. The main difference with the previous approach is that in order not to disrupt the natural dynamics of the network, annealing was not used (equivalent to setting *T* = 0 throughout the optimization). In other words, the algorithm was deterministic in the sense that changes in connectivity were only accepted if they diminished the cost function. The resulting greedy optimization scheme, however, often failed to escape local minima. We found that the relaxation of the performance criterion (90% of patterns retrieved instead of 100%) gave enough flexibility for the algorithm to escape these local minima and actually achieve an overall performance similar to the one obtained with simulated annealing. Therefore, we defined the variable *p*_*eff*_ as the effective number of patterns the network could successfully retrieve, from a total amount of *p*, where *p*_*eff*_ > 0.9*p*.

**Table:** **Algorithm 1 Online algorithm**.

1: $\{\bar{\bar{C}}\in {\mathbb{R}}^{N\times N}$ / $\overset{N}{\underset{j=1}{\sum }}C_{ij}=c_{0}$; *C*_*ii*_ = 0; 1 ≤ *i* ≤ *N*} ⊳ Generate random connectivity matrix
2: $p=p_{c}^{rand}$
3: $W_{ij}=\overset{p}{\underset{\mu =1}{\sum }}\xi_{i}^{\mu }\xi_{j}^{\mu }$ ⊳ Store $p=p_{c}^{rand}$ random patterns $\xi_{i}^{\mu }$
4: Compute *p*_*eff*_
5: while *p*_*eff*_>0.9·*p* **do**
6: *p* = *p*+10
7: $W_{ij}=\overset{p}{\underset{\mu =1}{\sum }}\xi_{i}^{\mu }\xi_{j}^{\mu }$
8: Compute *p*_*eff*_
9: while *p*_*eff*_ ≤ 0.9·*p* **do**
10: for *i* = 1:*N* **do** ⊳ This **for** can be done in parallel
11: Compute $E_{i}^{\epsilon }\left[{\bar{C}}_{i}\right]$ ⊳ Neuron *i* local energy
12: for *k* = 1:10 **do**
13: ${\bar{C}}_{i}^{f}={\bar{C}}_{i}$ ⊳ Dummy connectivity
14: Select randomly $C_{ij}^{f}$ such that *j*≠*i*
15: $C_{ij}^{f}=1-C_{ij}^{f}$ ⊳ Assess corresponding change
16: Compute $E_{i}^{\epsilon }\left[{\bar{C}}_{i}^{f}\right]$
17: if $E_{i}^{\epsilon }\left[{\bar{C}}_{i}^{f}\right]-E_{i}^{\epsilon }\left[{\bar{C}}_{i}\right]<0$ **then**
18: $E_{i}^{\epsilon }\left[{\bar{C}}_{i}\right]=E_{i}^{\epsilon }\left[{\bar{C}}_{i}^{f}\right]$
19: ${\bar{C}}_{i}={\bar{C}}_{i}^{f}$
20: end **if**
21: end **for**
22: end **for**
23:
24: if stopping condition is met **then**
25: break while
26: end **if**
27:
28: end **while**
29: end **while**

Given *N* neurons and *c*_0_ connections per neuron, we constructed the network's initial random connectivity matrix following the steps detailed before in Section 2.2. Then, an initial number *p*_0_ of patterns was loaded, equal to its maximum memory capacity, following Equation (3). Given these initial conditions, we alternated the optimization of the network connectivity and the loading of 10 new patterns. Given neuron *i*, a pre-synaptic neuron *j* was randomly selected using *randi*(*N*−1) function. If a connection existed for this combination of pre- and post-synaptic neurons (*C*_*ij*_ = 1), the effect of eliminating the connection was assessed, and inversely the effect of adding the connection was assessed if *C*_*ij*_ = 0. In both cases, the modification of *C*_*ij*_ was kept only if it reduced the local energy. This trial-and-error sequence was repeated 10 times for each neuron, after which pattern stability was tested. If more than 90% of patterns were successfully retrieved, a new set of 10 patterns was loaded. Otherwise, the optimization of *C*_*ij*_ was repeated until the retrieval condition was met. The algorithm stopped whenever the network's connectivity matrix did not vary for 50 consecutive repetitions. Since the memory load was no longer fixed, we set the parameter ϵ to a constant value throughout the optimization. In Section 3, we fixed ϵ = *N*/2.

## 3. Results

### 3.1. Noise reduction

#### 3.1.1. Storage capacity

**Figure 1:**
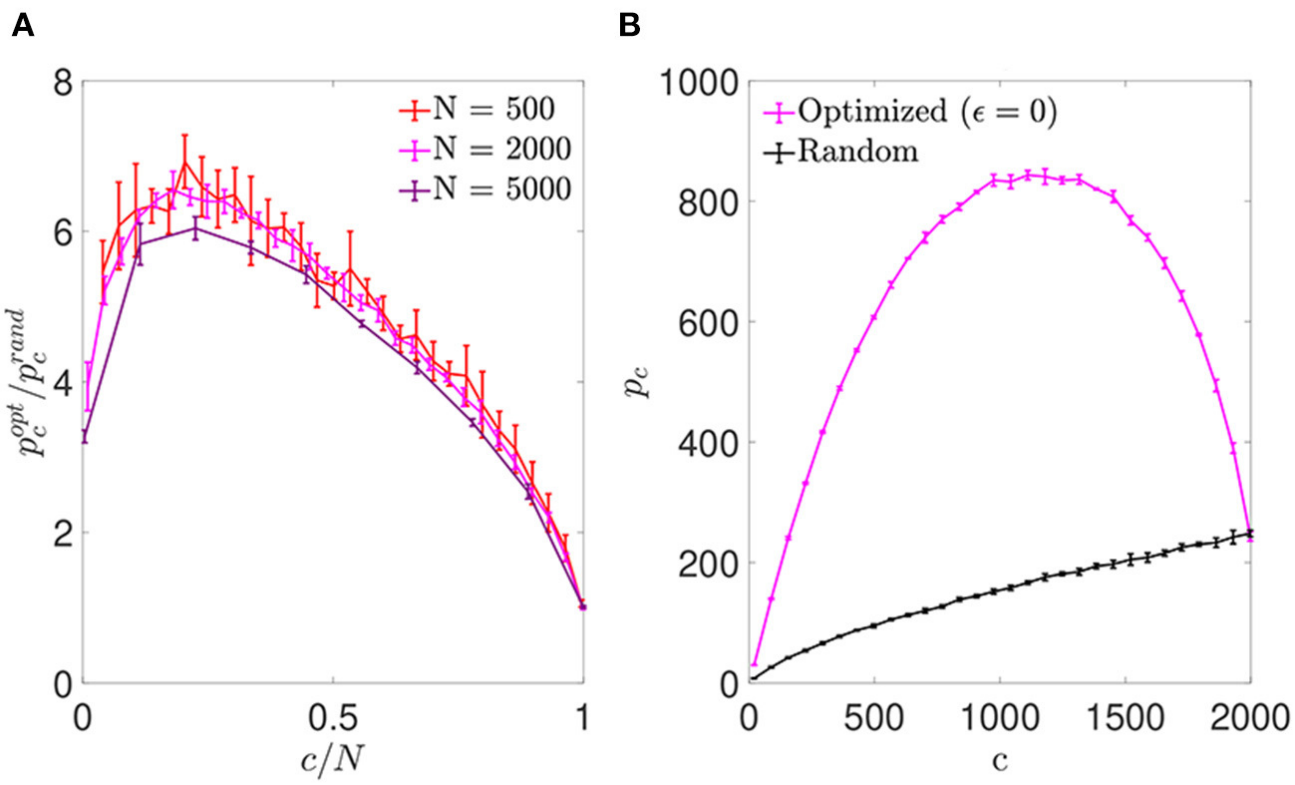
Networks optimized by noise reduction outperform randomly connected ones in terms of storage capacity. **(A)** Ratio between the storage capacity for optimized ($p_{c}^{opt}$) and random ($p_{c}^{rand}$) connectivity as a function of the number of connections per neuron for networks of different *N* (color coded; mean ± s.d.). **(B)** Overall maximum number of patterns (not normalized by *c*) that can be stored and retrieved in an optimized (magenta) or random (black) network of *N* = 2, 000 as a function of *c* (mean ± s.d.). Note that the maxima for $p_{c}^{opt}/p_{c}^{rand}$ **(A)** and $p_{c}^{opt}$ **(B)** occur for different connectivity levels, implying that more patterns can be stored using a higher number of less efficient connections. Pool of data corresponding to five simulations for each connectivity.

We first simulated networks of different size, with a number of neurons *N*∈{500; 2,000; 5,000} and a number of pre-synaptic connections per neuron covering the full range from highly diluted to fully connected. In each simulation, the connectivity matrix was optimized by implementing the simulated annealing algorithm, which minimized the cost function $E_{i}^{0}$ for each neuron (Equation 6). As a result, we obtained an increment in the storage capacity for all values of *N* and *c*. We plotted the ratio between the maximum number of patterns stored by optimized vs. random networks in otherwise identical settings, $p_{c}^{opt}/p_{c}^{rand}$ (Figure 1A). This ratio peaked for a connectivity around *c*/*N* ~ 0.20, independently of the network size, with a storage capacity for optimized networks up to seven-fold higher than the capacity for random networks. For more diluted networks the factor of improvement over random networks decreased. In the highly diluted extreme, *c*/*N* → 0, the improvement ratio tended to 3, while in the opposite limit *c*/*N* → 1 it tended to 1, which can be explained by the absence of degrees of freedom left for the optimization to proceed.

For simulations of increasing *c* and fixed *N*, the optimization tended to break the monotonic dependence between *p*_*c*_ and *c* found in random networks (where *p*_*c*_ is maximal for fully connected networks; Figure 1B). We found that the maximum number of stored patterns (not normalized by *c*) peaked at a connectivity near *c*/*N* ~ 0.59, different from the connectivity for which the improvement ratio maximized. This could be explained by a compromise allowing to maximize the overall amount of stored information by including a greater number of less efficient connections.

#### 3.1.2. Structural connectivity analysis

**Figure 2:**
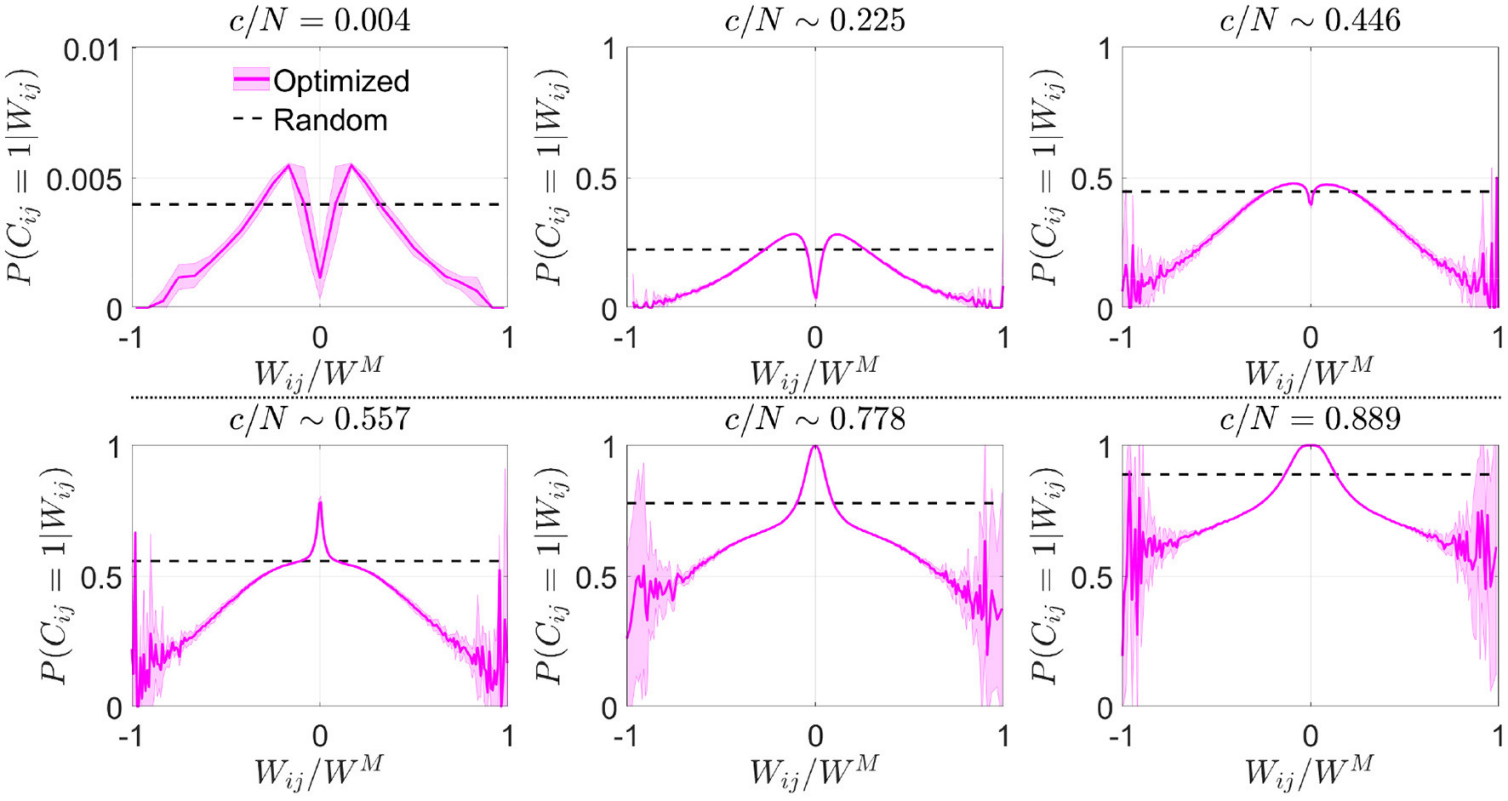
Optimization by noise reduction avoids connections with high absolute synaptic weight. Distribution of the conditional probability of having a connection between two neurons given their associated Hebbian weight for optimized (magenta; mean ± s.d.; *N* = 5,000) or random (dashed black; theoretical mean) networks. Each panel corresponds to a different connectivity value (indicated). Note that in some cases fluctuations are observed close to $|W_{ij}/W^{M}|\sim 1$, which are caused by a low number of available data points for extreme values of synaptic weight. Pool of data corresponding to five simulations for each connectivity.

We estimated from the resulting connectivity matrices the conditional probability distribution that, given the synaptic weight *W*_*ij*_, neurons *i* and *j* were connected in the optimized scheme. In a network with random connectivity, this probability is independent of *W*_*ij*_ because by definition $P\left(C_{ij}=1\right)=\frac{c}{N-1}$, but this was no longer the case in optimized networks. To improve visual comparison of results obtained for different *c* and *p*, we plotted this conditional probability *P*(*C*_*ij*_ = 1|*W*_*ij*_) vs. $W_{ij}/W^{M}$ (Figure 2), which represents the available weights normalized by the maximum absolute value reached by the synaptic weight matrix,


(8) $$\begin{array}{r} W^{M}=\underset{\begin{array}{c} i\neq j\\ 1\leq i,j\leq N \end{array}}{\max}\left\{\left|W_{ij}\right|\right\}. \end{array}$$

Note that *W*^*M*^ is not necessarily equal to *p*, the maximum theoretical absolute value for *W*_*ij*_. The probability that a given *W*_*ij*_ takes a value *p* is equivalent to the probability of success in *p* consecutive coin flips, which becomes increasingly unlikely as *p* grows.

In comparison with the uniform distribution, we observed that the optimized network in the sparse connectivity region tended to favor synapses within a range of low weight values, avoiding those with extreme high or low absolute weight (Figure 2). As connectivity increased, the network started to make use of close-to-zero weights, while still avoiding synapses with high absolute weight. After reaching the connectivity region near *c*/*N* ~ 0.59 (where pattern storage peaked), a sudden boost in the use of connections with close-to-zero weight was observed. We speculate that the reason behind this behavior is that optimization at this point minimizes the contribution of new connections to the local field. Eventually, synapses with high absolute weight were included, but always with a probability lower than in random networks.

#### 3.1.3. Signal-to-noise analysis

**Figure 3:**
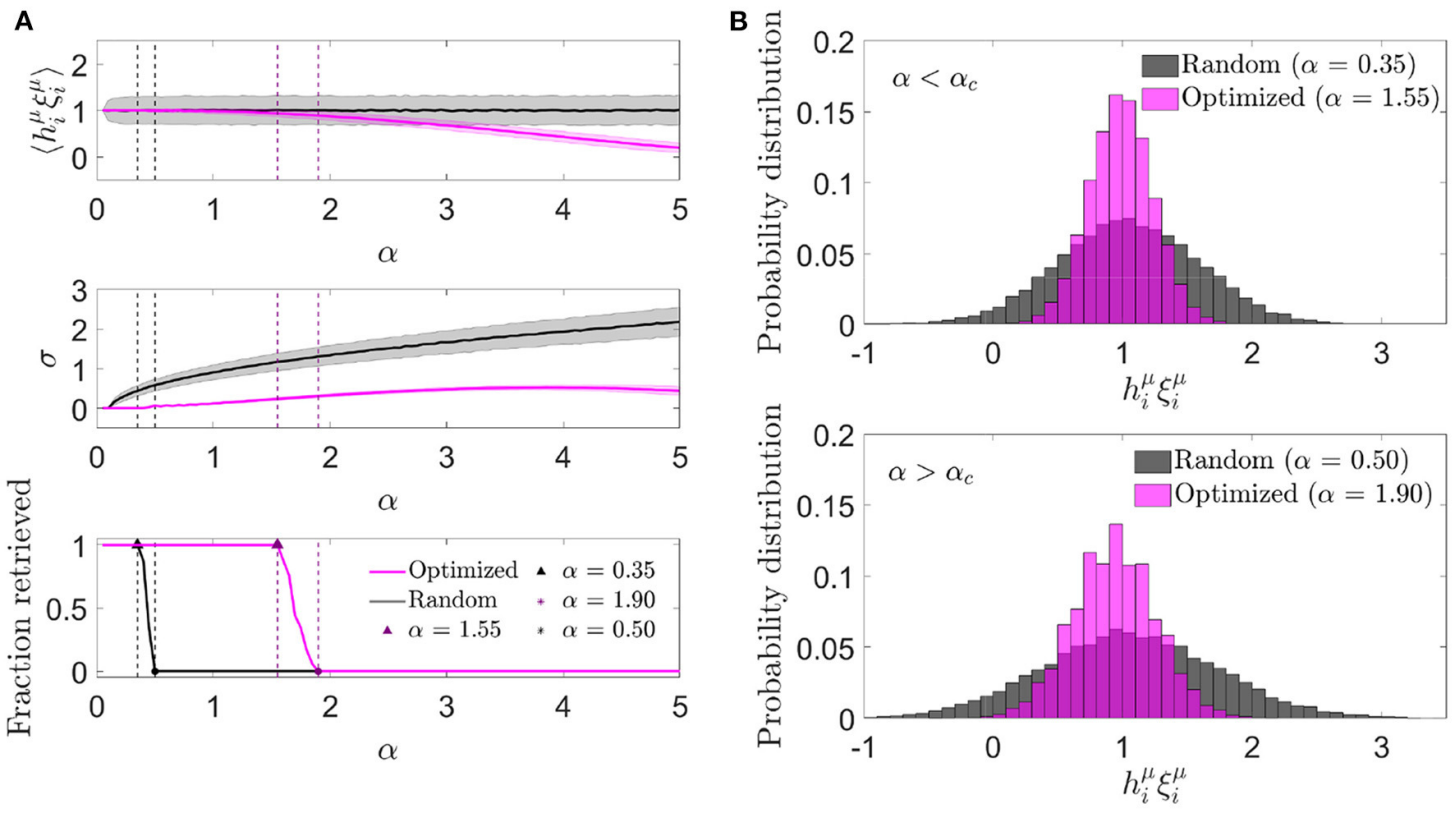
Noise in optimized networks has a reduced variability at the expense of a negative mean. **(A)** One representative simulation distribution across neurons of the average (top) and standard deviation (center) of the aligned local field (mean ± s.d.), together with the fraction of retrieved patterns (bottom) as a function of the memory load, for optimized (magenta) or random (black) networks. **(B)** Distribution of the aligned local field for specific memory loads slightly lower (top) and higher (bottom) than the storage capacity [indicated in **(A)**] for both kinds of network (same color code). All plots correspond to networks with *N* = 2, 000 and *c* = 20.

We next characterized the aligned local field distribution ($h_{i}^{\nu }\xi_{i}^{\nu }$) in optimized networks. Given that the heuristic minimization of $E_{i}^{0}$ never took the cost function to exactly zero, neurons received, on average, a non-zero noise contribution, expected to be lower than in randomly connected networks. We analyzed this distribution for different memory loads in a network with fixed *c* and *N* (Figure 3A). In contrast to randomly connected networks, the mean aligned field tended to decrease with α, which implied that the optimization found it convenient to anti-correlate the noise and the signal as a means of minimizing $E_{i}^{0}$. In spite of this anti-correlation, retrieval was possible for a larger range of α values than what was observed with otherwise identical random networks.

We next studied the standard deviation of the local field to understand if a decrease in variability was compensating for the a priori negative effect of a decrease in mean aligned local field. We observed that, indeed, the increment in the storage capacity could be explained by a substantially narrower noise distribution than the one obtained with random connectivity (Figure 3B). This suggests that fluctuations of the noise term were small enough to let the network store patterns beyond the classical limit, even if they occurred around a negative mean.

The unexpected finding that, in order to minimize the overall noise, the optimization tended to decrease the mean aligned field to values lower than 1 (implying a negative mean aligned noise), led us to ask if better cost functions would lead to a situation in which the noise reinforced the signal, thus improving the overall performance of the network. We explore this possibility in Section 3.2.

#### 3.1.4. Basin of attraction

**Figure 4:**
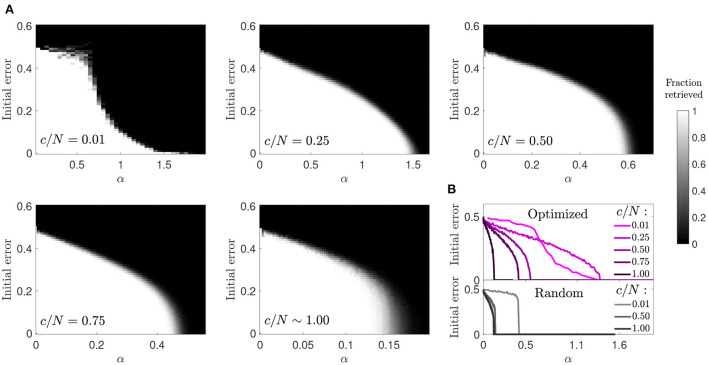
Reduced basin of attraction in optimized networks with diluted connectivity. **(A)** Fraction of patterns that a network of *N* = 2, 000 can successfully retrieve (gray-scale), relative to the initial error. **(B)** Phase transition curves from **(A)** condensed in a single plot (top) together with similar curves corresponding to a random network (bottom). Connectivity is color coded.

We next studied the basin of attraction in optimized networks to understand if an increase in storage capacity came at the cost of a reduction in attractor strength (Figure 4). We observed that for most connectivity levels the basin of attraction was only moderately reduced. This reduction was comparable to the one observed in random networks, although the transition to the amnesic phase was smoother. The highest contrast with the behavior of random networks was observed in the case of diluted optimized networks (*c*/*N* = 0.01), where large basins of attraction were observed for values of the memory load α up to around 0.7, above which they tended to be very small. Close to the storage capacity limit, although α was high, the attractors were very weak, as a pattern could only be retrieved if the initial state was almost identical to it. However, for *c*/*N* = 0.25, a connectivity within the region where the maximum improvement ratio in storage capacity was achieved (Figure 1), attractors were more robust than in diluted optimized networks, and only slightly less robust than in random networks at a comparable distance from the critical storage capacity.

The increase in the size of the basin of attraction from *c*/*N* = 0.01 to *c*/*N* = 0.25 in optimized networks is counter intuitive, but analytic modeling of networks with random connectivity provides a potential explanation (Roudi and Treves, 2004). In a non-diluted, random connectivity network, the field of a given neuron has a noise term proportional to its activity, caused by feedback of the neuron's activity state through multiple-synaptic loops. The probability of existence of such connectivity loops, starting in the neuron and projecting back to it, decreases toward zero in diluted networks, and so thus the effect of this noise term. It is possible that the optimized network leverages from multi-synaptic feedback loops to increase the robustness of attractors, as the sudden decay in the size of the basin of attraction but not in overall storage capacity from *c*/*N* = 0.25 to *c*/*N* = 0.01 seems to suggest.

### 3.2. Signal reinforcement

#### 3.2.1. Storage capacity

**Figure 5:**
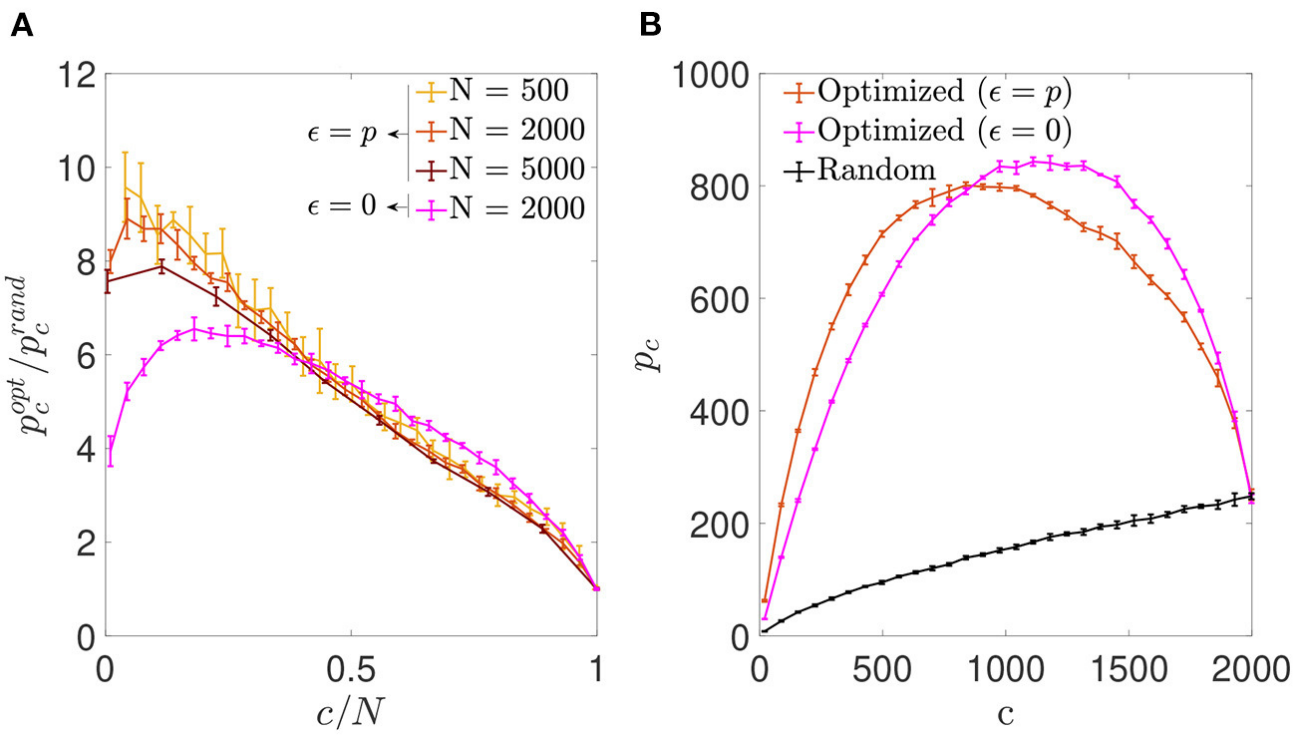
Signal reinforcement outperforms noise reduction and is optimal with highly diluted connectivity. **(A)** Ratio between the storage capacity for optimized ($p_{c}^{opt}$) and random ($p_{c}^{rand}$) connectivity as a function of the number of connections per neuron for networks of different *N* (shades of red; mean ± s.d.). For comparison, the curve corresponding to noise reduction is included (magenta; *N* = 2, 000). **(B)** Overall maximum number of patterns (not normalized by *c*) that can be stored and retrieved as a function of *c* in a network with *N* = 2, 000 optimized by signal reinforcement (orange), optimized by noise reduction (magenta) or with random connectivity (black) (mean ± s.d.). Note that, as in Figure 1, the maxima for $p_{c}^{opt}/p_{c}^{rand}$ **(A)** and $p_{c}^{opt}$ **(B)** occur for different connectivity levels. Pool of data corresponding to five simulations for each connectivity.

Inspired by the results in the previous section, we next assessed the possibility of optimization by reinforcement of the signal rather than noise reduction, by using the same optimization procedure with a different cost function (Equation 7). We observed that, as in the previous section, the optimization process increased the storage capacity of autoassociative networks (Figure 5A). However, in contrast to the ϵ = 0 case, the improvement ratio $p_{c}^{opt}/p_{c}^{rand}$ for the optimization with ϵ = *p* increased monotonically with network dilution for most of the *c*/*N* range, reaching its best performance $p_{c}^{opt}/p_{c}^{rand}\sim 10$ around the lowest connectivity values.

We also observed that the overall number of patterns *p* that a network of fixed *N* could store and retrieve was higher if optimized by minimizing $E_{i}^{p}$ than by minimizing $E_{i}^{0}$ in the low connectivity range (Figure 5B), but the opposite was true for high connectivity. While the connectivity capable of storing more patterns for the optimization with ϵ = 0 was near *c*/*N* ~ 0.59, for the optimization ϵ = *p* it was *c*/*N* ~ 0.42, with slightly fewer patterns in total. However, both of these limits are far from the biologically plausible limit of high dilution, where signal-reinforcing networks seem to outperform noise-minimization ones.

**Figure 6:**
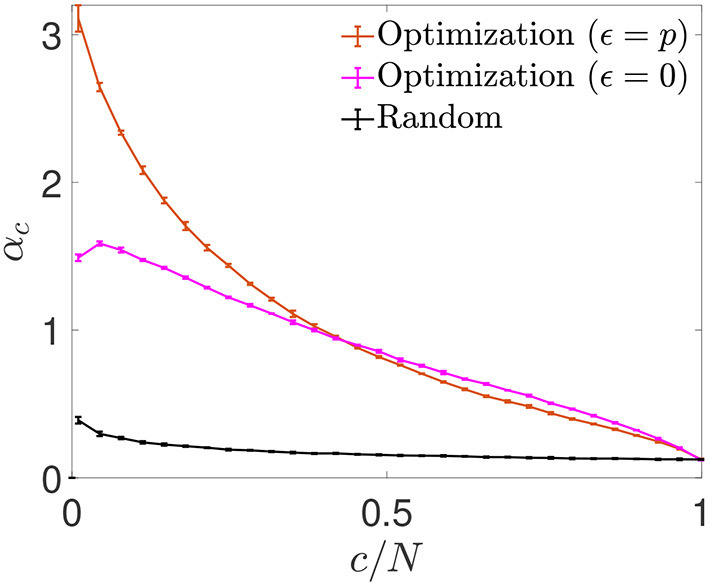
Storage capacity of optimized and random networks. Critical storage capacity vs. connectivity ratio for networks (*N* = 2, 000) with random connectivity (black) or connectivity optimized by signal reinforcement (orange) or noise reduction (magenta) (mean ± s.d.). Pool of data corresponding to five simulations for each connectivity. *T*-test comparisons for data in the lowest connectivity values show significant differences between the storage capacity of random networks and that of noise reduction (*p*-value: 10^−6^) or signal reinforcement (*p*-value: 10^−7^).

We next plotted the α_*c*_ curves as a function of *c*/*N* (Figure 6), which allowed for a better visualization of the fact that for a connectivity of *c*/*N* = 0.01, both optimizations achieved an enhanced storage capacity, in the case of signal-reinforcement networks up to an order of magnitude greater than obtained using random connectivity. When minimizing $E_{i}^{0}$, the capacity was close to α_*c*_ ~ 1.49, while in $E_{i}^{p}$ minimization, the critical capacity more than doubled the latter, reaching α_*c*_ ~ 3.15.

#### 3.2.2. Structural connectivity analysis

**Figure 7:**
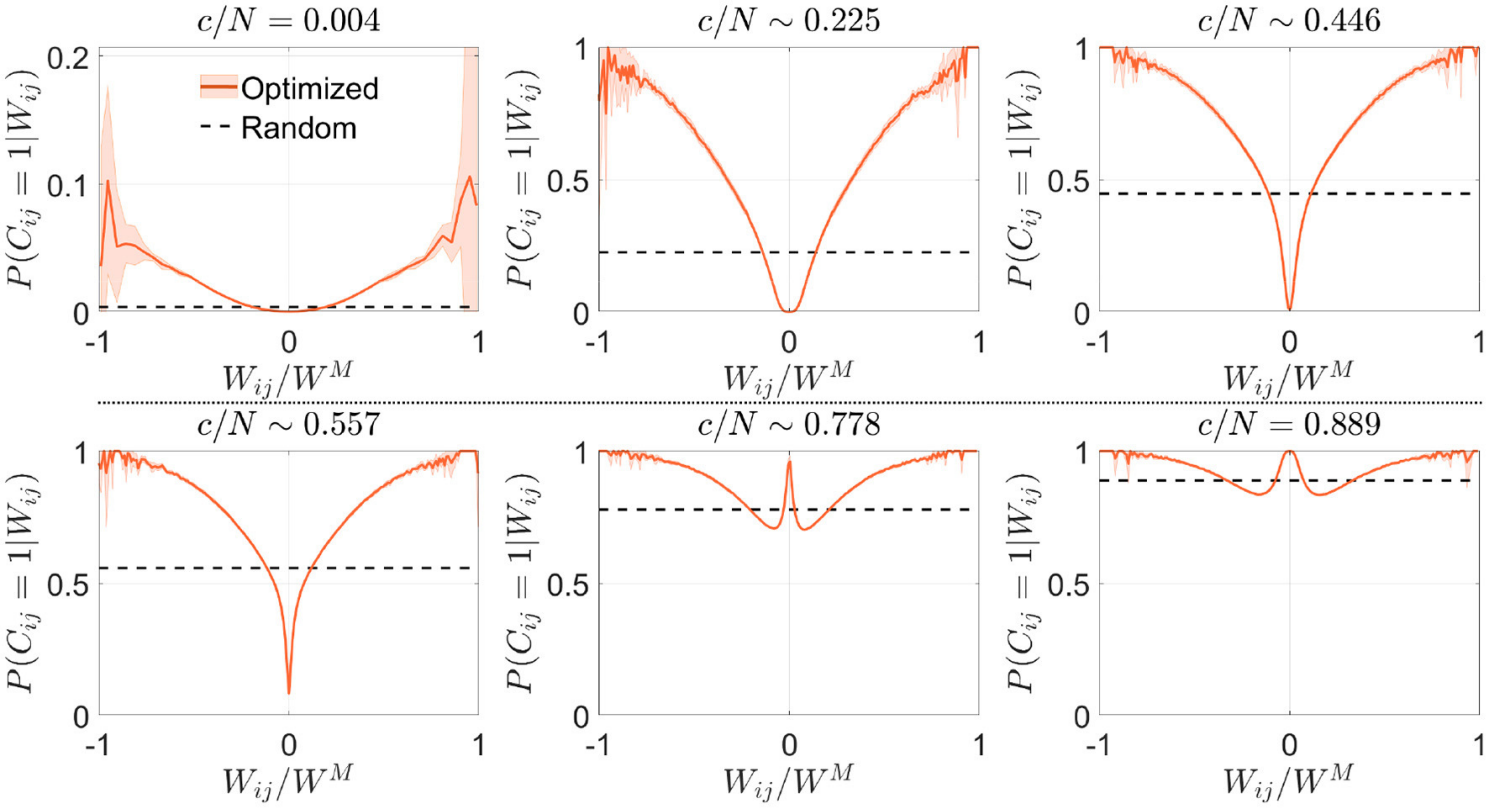
Optimization by signal reinforcement preferentially selects connections with high absolute synaptic weight. Distribution of the conditional probability of having a connection between two neurons given their associated Hebbian weight for networks optimized by signal reinforcement (orange; mean ± s.d.; *N* = 5, 000) or with random connectivity (black; theoretical mean). Each panel corresponds to a different value of the connectivity (indicated). Note that fluctuations are observed near $|W_{ij}/W^{M}|\sim 1$ due to low sampling, as in Figure 2. Pool of data corresponding to five simulations for each connectivity.

We next asked if the criteria for selecting synapses in the signal reinforcement scenario were similar to those found in the previous section. We observed that the conditional distribution *P*(*C*_*ij*_ = 1|*W*_*ij*_) obtained in the low connectivity range increased monotonically with the absolute value of the synaptic weights, implying that, in contrast to what was previously observed, this time the optimization process favored connections with high absolute weight (Figure 7). As connectivity increased, networks tended to use all available synapses with high absolute value, which forced them to include lower modulus synapses. For an intermediate range of connectivity (>*c*/*N* ~ 0.42, where pattern storage peaked), close-to-zero weight synapses started being used, similarly to what happened in the noise reduction scenario. For highly connected networks (*c*/*N*≳0.75), the pool of available close-to-zero synapses also depleted and more intermediate-range synapses were included. This strategy of selecting synapses with a high degree of consensus across patterns seems opposite to the one described for noise reduction.

#### 3.2.3. Signal-to-noise analysis

**Figure 8:**
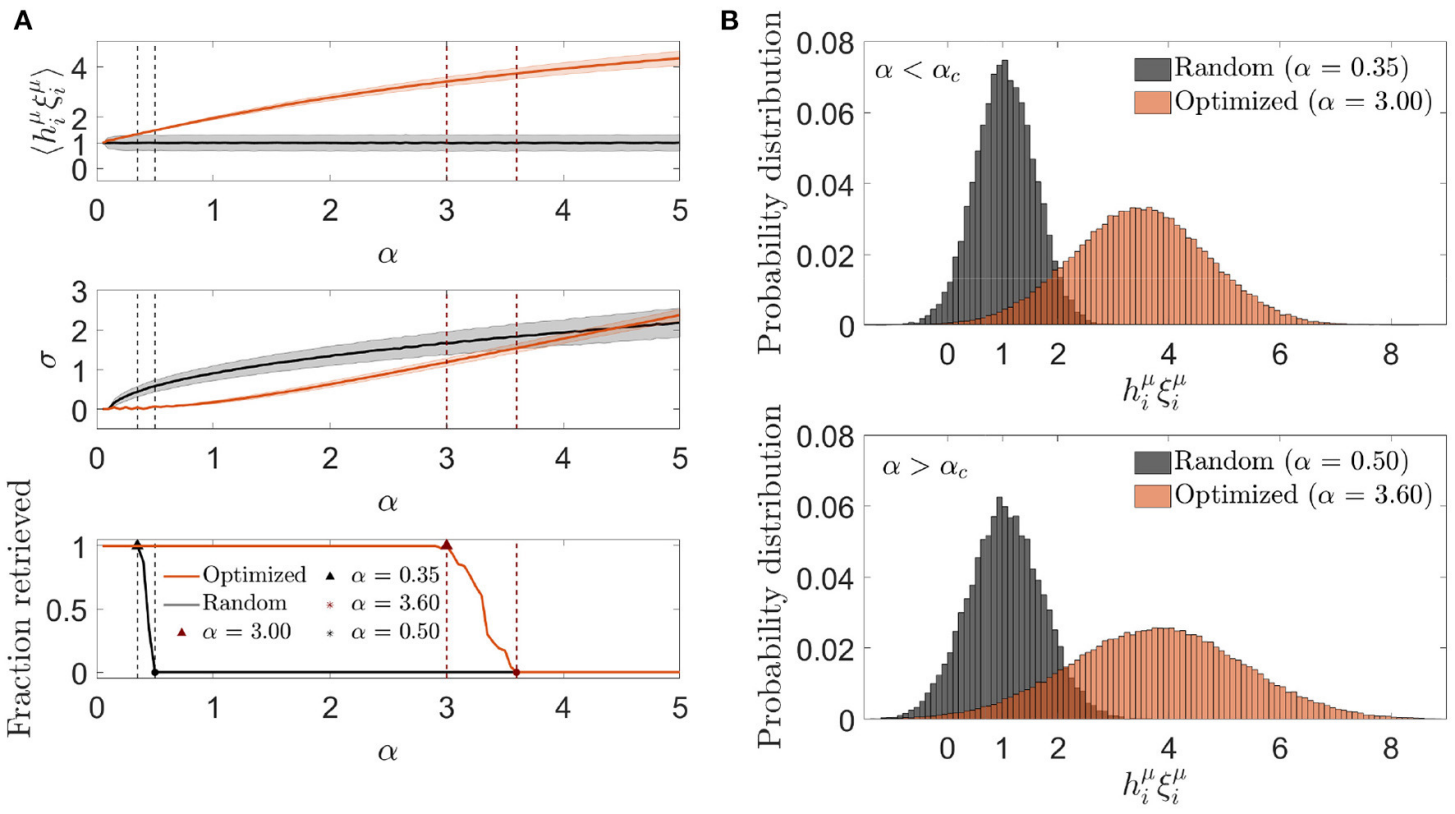
Optimization by signal reinforcement results from a mean noise increasing faster than its variability. **(A)** One representative simulation distribution across neurons (of only one trial simulation) of the average (top) and standard deviation (center) of the aligned local field (mean ± s.d.), together with the fraction of retrieved patterns (bottom) as a function of the memory load, for networks with a connectivity that is optimized by signal reinforcement (color) or random (black). **(B)** Distribution of the aligned local field for specific memory loads slightly lower (top) and higher (bottom) than the storage capacity (indicated in **A**) for both kinds of network (same color code). All plots correspond to networks with *N* = 2, 000 and *c* = 20.

As previously, we plotted the mean and standard deviation of the aligned local field as a function of the memory load α (Figure 8). We observed that the mean aligned local field increased monotonically with α, as expected from the fact that the cost function was designed to make the noise reinforce the signal, reaching a substantial deviation from 1, its zero-noise value. The standard deviation also increased with α, although it remained lower than the one corresponding to an otherwise identical random network for values of α where retrieval was possible. At α_*c*_, the standard deviation for the optimized network was noticeably higher than the one corresponding to the random network considered at its own α_*c*_ value, implying that the increase in storage capacity was due to an ability to make the mean aligned local field increase faster than its standard deviation for a limited range of values of α.

#### 3.2.4. Basin of attraction

**Figure 9:**
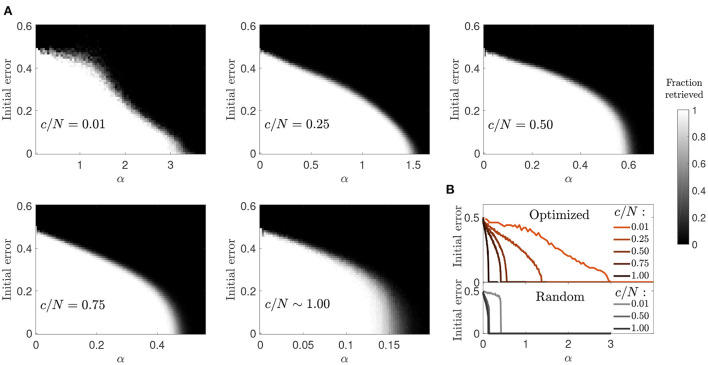
Enhanced basins of attraction in networks optimized by signal reinforcement. **(A)** Fraction of patterns (gray-scale) a network of *N* = 2, 000 can successfully retrieve, after initializing it in each of the α·*c* stored patterns with a given initial error. **(B)** Condenses shown information for each connectivity (color coded) by plotting the maximum initial error tolerance for which the network can successfully retrieve all patterns for each α value (optimized networks, top; random networks, bottom).

Similar to the previous optimization, and in contrast to random connectivity networks, the basin of attraction decreased progressively rather than abruptly with memory load (Figure 9). However, when comparing the two optimization strategies, we observed that memories in the ϵ = *p* scenario were more robust in the connectivity range *c*/*N*≲0.4. This indicates that the new optimization strategy was better than the previous one due to a synergistic combination of increased storage capacity and larger basins of attraction. As connectivity increased, the basins of attraction tended to look like the ones observed in random networks, as in the previous optimization case.

### 3.3. Online algorithm

To simulate a situation similar to the development of the human brain, where connectivity changes as the subject learns, we explored an online learning scenario with dynamic generation and elimination of synapses. Results shown in this section correspond to simulations of *N* = 2000 networks with different initial random connectivity values *c*_0_. The connectivity optimization was done by implementing Algorithm 1 with the cost function shown in Equation (7), and setting ϵ = *N*/2.

#### 3.3.1. Mean connectivity variation

**Figure 10:**
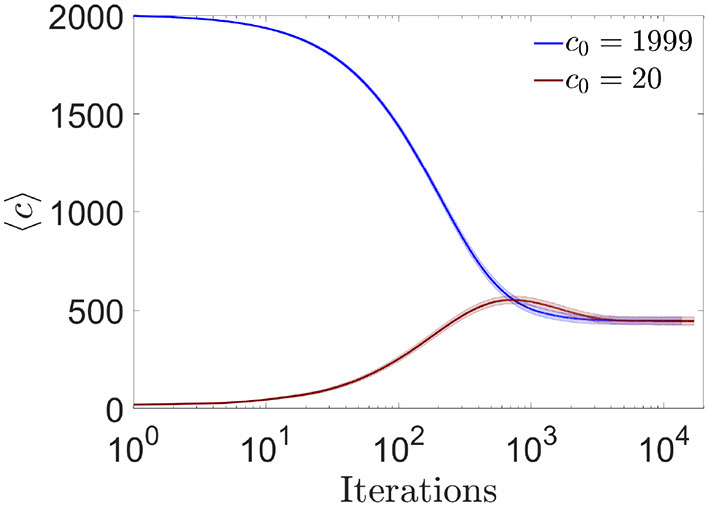
Evolution toward a common equilibrium connectivity for the online algorithm. Distribution of the number of pre-synaptic connections per neuron across iterations of the online algorithm, for networks with *N* = 2, 000, fully connected (blue) or highly diluted (brown) initial connectivity (mean ± s.d). During one iteration, 10 random changes in pre-synaptic connectivity were evaluated (one at a time) for each neuron (i.e., the complete loop over all neurons, *for i* = *1:N*, in Algorithm 1). Each curve corresponds to one simulation trial.

We first studied the evolution of the mean number of connections per neuron along the learning process. As previously mentioned, the algorithm allowed each neuron to freely eliminate or generate connections, with minimization of their own cost function as the only constraint. We asked if the final number of connections, which was a priori unknown, depended on initial conditions (for example on the initial connectivity value *c*_0_) or on the history of the learning process. We observed that the final average connectivity did not depend on initial conditions, stabilizing at a value *c*_*est*_ ~ 445 for all tested *c*_0_ (this stability value depended on ϵ) (Figure 10). For networks with initial connectivity above *c*_*est*_ the average connectivity decreased monotonically, i.e., pruning predominated throughout the learning process. For networks with initial connectivity below *c*_*est*_ we observed a dynamic qualitatively analogous to the maturation of the human brain, with an initial creation-predominant period and a late pruning-predominant period.

#### 3.3.2. Storage capacity

**Figure 11:**
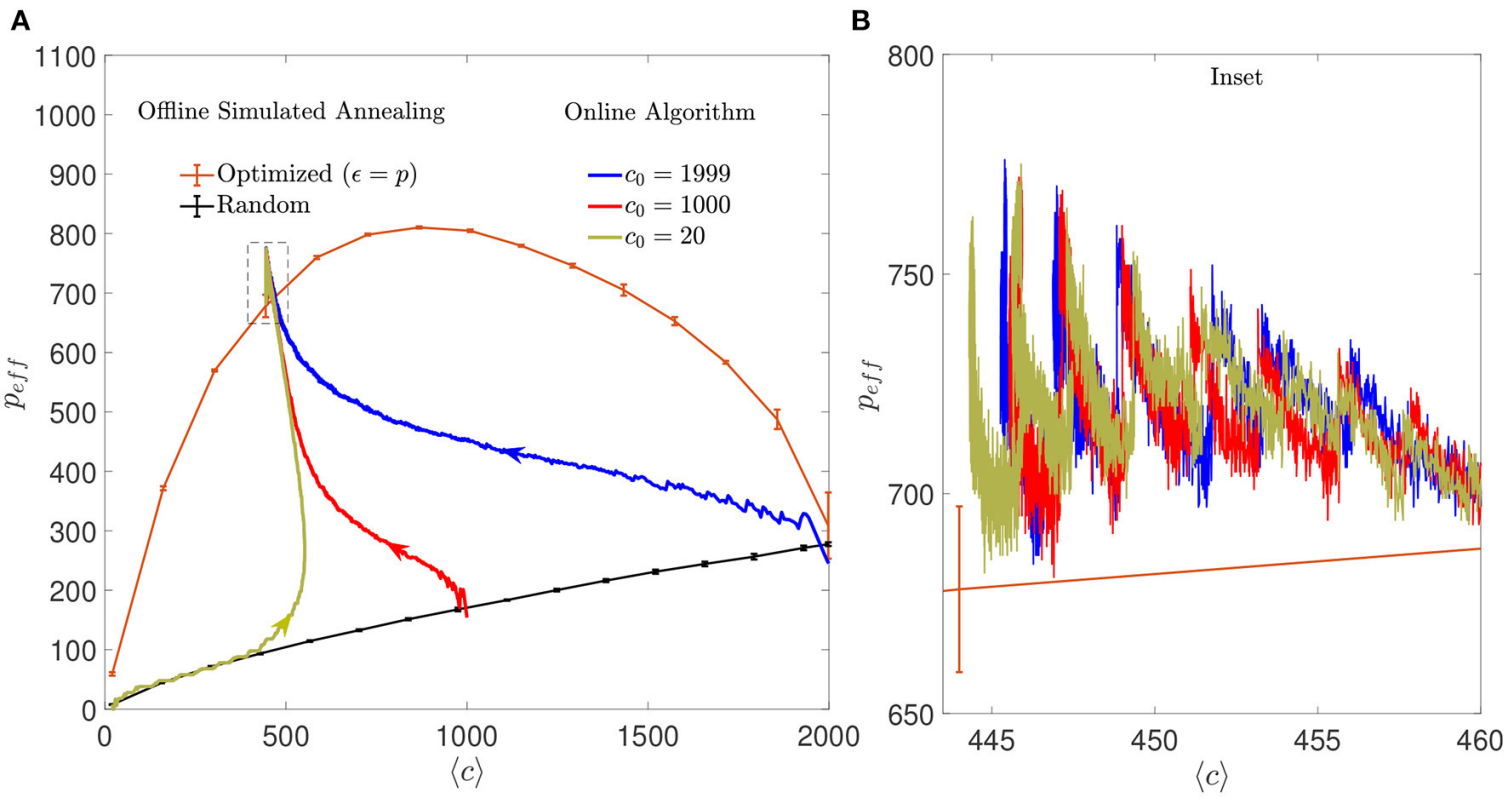
Regimes dominated by addition or pruning of connections lead to a performance similar to the one corresponding to offline optimized connectivity. **(A)** Evolution of the learning process of the online algorithm in the space of effective maximum number of patterns vs. mean connectivity for different initial conditions: highly diluted (dark yellow), intermediate (red), or fully connected (blue) (a single simulation for each initial condition is shown). Arrows indicate the direction in which the networks evolved from the corresponding initial conditions. For comparison, the distribution of maximum *p*_*eff*_ that offline optimized (orange) or random (black) networks can achieve with similar criteria is also shown (mean ± s.d., computed over five simulations each). Note that the final network optimized by the online algorithm performs slightly better, possibly because the number of connections is not fixed across neurons. Dashed box corresponds to inset shown in **(B)**. All networks have *N* = 2, 000. **(B)** Inset of **(A)** showing in detail the last iterations of the online algorithm for the three different initial conditions. Every time a set of 10 patterns is loaded to the network, the effective storage capacity drops abruptly. Within the following iterations, *p*_*eff*_ increases until 90% of patterns are retrieved or the simulation stops.

The online algorithm substantially increased the storage capacity of the autoassociative networks compared to what would be obtained without modification of the connectivity matrix. The improvement was comparable to the one obtained with the offline simulated annealing algorithm $E_{i}^{p}$ (Figure 11). As observed with mean connectivity, the final effective storage capacity reached by the network did not depend on the initial connectivity, although it was strongly dependent on the ϵ parameter. Interestingly, when the initial connectivity was set to highly diluted, the initial increase in *p*_*eff*_ occurred along the storage capacity limit for random networks, implying that the optimization was similar to what would be obtained by adding random connections, up to a connectivity level close to the final one. From that point on, optimization proper took place, and the optimized *p*_*eff*_ increased above the random limit in a process initially dominated by addition and later on by pruning. In contrast, the effect of selective pruning was never equivalent to that of a random one. Given the monotonically increasing relationship between *p*_*c*_ and *c* in random networks, random pruning necessarily results in a decay in *p*_*c*_.

Put together, these results suggests that the algorithm was able to converge to a connectivity configuration that represented a global minimum in the space of solutions, with a unique intermediate dynamics that depended on initial conditions.

## 4. Discussion

Our main result is that it is possible to improve the storage capacity of an autoassociative network by optimizing its connectivity matrix. We found that if structural connectivity is optimized to minimize the noise present in the local field of each neuron, up to a seven-fold improvement in the storage capacity can be obtained in comparison to random networks. This maximal improvement, however, occurs with a relatively dense connectivity close to *c*/*N* ~ 0.20, which is not typical in the mammalian brain. We next found that an even stronger improvement could be obtained by reinforcing the signal rather than minimizing the noise, this time reaching almost one full order of magnitude in the biologically plausible highly diluted limit. These results suggest that diluted autoassociative networks would benefit most from a mechanism where the connectivity configuration allows the noise term to reinforce the signal. The fact that all networks studied in this work have a similar matrix of synaptic weights, governed by the Hebbian principle, and yet levels of performance that span over one order of magnitude, stresses the importance of considering the remodeling of network architecture as a process potentially independent and complementary to synaptic weight modification.

We found that in the diluted connectivity region, the signal-reinforcement optimization was achieved by selecting connections with high consensus across patterns, with a probability that decreased with the modulus of the synaptic weight. Our results are in the same direction of previous work showing a substantial storage capacity increase in a highly diluted network that only retains the *c* strongest connections per neuron (Montemurro and Tamarit, 2001). This *extreme pruning* scheme was shown analytically to make the storage capacity diverge in the limit $c\ll \min\left\{\frac{N}{2^{p-1}},\ln\left(N\right)\right\}$, which, however, is too diluted for real brains. Simulations under the more plausible constraint of *c* ≪ *N* resulted in a storage capacity smaller but in the range of the one we have found. This is consistent with our observation that, although optimized networks tend to prefer high absolute weights, they do not include only the highest ones, and instead recruit as well a number of intermediate weights. More generally, the idea of a connectivity scheme that reinforces the Hebbian principle was presented in the cited work and is also part of our results using the signal-reinforcement optimization.

Our model used rather simplistic units and architecture. Similar results could perhaps be replicated in models that include more realistic elements such as networks with graded response units and non binary patterns (Treves and Rolls, 1991). We speculate that graded response units may provide the network with one extra degree of freedom, since, for a given neuron, the demands for connectivity optimization could be weighted by its activity level in each stored pattern. Regarding architecture, since our network does not mimic the hierarchical organization of the brain (Fulvi Mari, 2004), it most suitably models phenomena taking place in short-range cortical networks, where remodeling of connections is most relevant and has been most extensively studied.

Brains do not have a fixed connectivity throughout the lifetime of humans and other mammals. Evidence indicates that connectivity levels increase during childhood, reaching a maximum around the age of 2, and then decrease, reaching a stable value in late adolescence or early adulthood (Huttenlocher, 1979; Lichtman and Colman, 2000; Hua and Smith, 2004; Navlakha et al., 2015), a period roughly coinciding with the one originating most of long lasting memories (Rubin et al., 1998). This implies that addition and pruning take place in parallel to the most substantial stages of learning. In a simplified model of this process, our online algorithm explores the evolution of connectivity and storage capacity in a network where neurons can freely add or delete connections as the network incorporates more patterns. We observed that if the network is initialized at a high connectivity level, it mainly prunes connections, with an immediate gain over random connectivity setups. However, if its initial connectivity is low, a process with several stages takes place. During the initial stage, connections are added with a gain in storage capacity equivalent to the addition of random connections, implying that a similar path would be followed by networks randomly initialized in a range of values of low connectivity. Equivalently, this first stage that could be associated with the initial development of the brain could add connections randomly instead of selectively, with a similar effect on performance. Once connectivity is close to the final one (unknown a priori but dependent on the parameter ϵ), the addition of connections becomes more meaningful, improving the storage capacity beyond the level of random networks. Remarkably, this process takes connectivity beyond the final value, reminiscent of what is actually observed during childhood in the human brain. In a third stage, increase in storage capacity is associated with a net loss of connections, until an equilibrium is reached and storage capacity is close to the global minimum of the cost function obtained with offline minimization. Although this dynamic is complex, the final state of the network is independent of initial conditions, suggesting a strong global minimum reached by the optimization process.

Throughout this work we have used an extremely simple model of autoassociative memory that, however, captures all the basic behaviors exhibited by this kind of network even in biologically plausible or experimental setups (Roudi and Latham, 2007; Carrillo-Reid et al., 2016). Using mean network activity of 50%, as in the original Hopfield model, helped making our simulations computationally efficient by keeping the storage capacity relatively low. The effects of connectivity optimization on sparser networks, however, with a biologically plausible value around 5 or 10%, should be explored in the future. One possibility is that connectivity optimization becomes less effective because the pool of active neurons from which to choose is more restricted. In contrast, we speculate that other modifications aiming to add biological plausibility could make connectivity optimization even more relevant than in the present work. For example, if memorized patterns were obtained from a distribution exhibiting a set of non-trivial correlations (Kropff and Treves, 2007), the initial stage of optimization could capture early the main statistical features of inputs, making corrections at a later stage less important or necessary. In addition, shared features across patterns might help in the reinforcement of the signal by the noise. It has been shown that in this scenario the selected deletion of neurons can yield a significant improvement in storage capacity, implying that a much more subtle correction in connectivity could lead to similar or higher performance levels (Kropff, 2007).

We have shown that in a simple setup, connectivity optimization can improve storage capacity, in some cases up to around one order of magnitude. Though considerable, this gain might still be not enough to explain the viability of attractor networks in the human brain, given all the drawbacks of biologically plausible networks. Further work on the effect of optimized connectivity in networks that include some of these details of biological plausibility could perhaps place our estimation of storage capacity at least in the reasonable range between thousands and tens of thousands of memories, a limit compatible with the number of faces we remember or the number of words we use in our daily life.

## Data availability statement

The datasets presented in this study can be found in online repositories. The names of the repository/repositories and accession number(s) can be found at: https://github.com/Facuemina/Selective-Connectivity-2022.

## Author contributions

FE and EK planned the simulations, analyzed the results, and wrote the manuscript. FE wrote the scripts and ran the simulations. Both authors contributed to the article and approved the submitted version.

## Funding

This work had support from PICT-2019-2596 (Science Ministry, Argentina) and Human Frontiers Science Program RGY0072/2018 (EK).

## Conflict of interest

The authors declare that the research was conducted in the absence of any commercial or financial relationships that could be construed as a potential conflict of interest.

## Publisher's note

All claims expressed in this article are solely those of the authors and do not necessarily represent those of their affiliated organizations, or those of the publisher, the editors and the reviewers. Any product that may be evaluated in this article, or claim that may be made by its manufacturer, is not guaranteed or endorsed by the publisher.
